# Beyond the Rankin Scale: A Patient‐Centered Score for Meaningful Recovery After Mechanical Thrombectomy in Older Adults With Pre‐Stroke Disability

**DOI:** 10.1111/ggi.70874

**Published:** 2026-10-16

**Authors:** Hiroyasu Inoue, Masahiro Oomura, Yoshitaka Nakamura, Yusuke Nishikawa, Yuto Uchida, Shoya Kusui, Atsuhito Taishaku, Yoshiro Nozaki, Tomoyasu Yamanaka, Teishiki Shibata, Yasuhide Inoue, Yoko Taniguchi, Yuta Madokoro, Toyohiro Sato, Teppei Fujioka, Masayuki Mizuno, Shoji Kawashima, Mitsuhito Mase, Noriyuki Matsukawa

**Affiliations:** ^1^ Department of Neurology Nagoya City University Graduate School of Medical Sciences and Medical School Nagoya Aichi Japan; ^2^ Department of Neurosurgery Nagoya City University Graduate School of Medical Sciences and Medical School Nagoya Aichi Japan; ^3^ Department of Radiology and Radiological Science Johns Hopkins University School of Medicine Baltimore Maryland USA

**Keywords:** acute ischemic stroke, mechanical thrombectomy, oldest‐old, pre‐stroke disability, quality of life

## Abstract

**Aim:**

The benefit of mechanical thrombectomy (MT) for acute ischemic stroke in patients with pre‐stroke moderate‐to‐severe disability is difficult to evaluate using the modified Rankin Scale (mRS) alone. We developed and evaluated the SMILE‐Stroke score as a patient‐centered outcome measure.

**Methods:**

We conducted a retrospective cohort study of 31 patients with pre‐stroke mRS scores of 3–5 who underwent MT. The 10‐point SMILE‐Stroke score, comprising Speech, Mobility, Ingestion, Lavatory, and Engagement domains, was assessed at discharge; a score ≥ 4 was prespecified as favorable. Interrater reliability, convergent validity with the Barthel Index, associations with discharge disposition and mRS, and pre‐stroke SMILE‐Stroke scores were evaluated.

**Results:**

Twelve patients (38.7%) achieved a favorable SMILE‐Stroke outcome. Interrater reliability was excellent for the total score (single‐rater intraclass correlation coefficient = 0.957) and dichotomized outcome (Fleiss' *κ* = 0.863). The score correlated strongly with the discharge Barthel Index (Spearman's *ρ* = 0.86, *p* < 0.001) and showed a graded association with discharge disposition (Kruskal–Wallis *p* = 0.0012). For distinguishing discharge to home/original residence or rehabilitation hospital from long‐term care hospital or death, the SMILE‐Stroke score yielded an AUC of 0.82. Notably, 6 of 19 patients (31.6%) with 90‐day mRS scores of 4–5 achieved a favorable SMILE‐Stroke outcome.

**Conclusions:**

The SMILE‐Stroke score was feasible, reproducible, and associated with established functional and discharge outcomes in selected MT‐treated patients with pre‐stroke disability. It may complement the mRS by identifying preserved or regained patient‐centered functional domains within poor mRS categories.

## Graphical abstract

The SMILE‐Stroke score is a brief, patient‐centered outcome measure for older adults with pre‐stroke disability who undergo mechanical thrombectomy. Nearly one‐third of patients with poor 90‐day modified Rankin Scale scores of 4–5 achieved a favorable SMILE‐Stroke outcome, revealing preserved communication, oral intake, and engagement missed by the Rankin Scale.

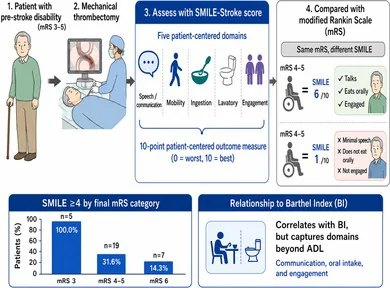

## 1 Introduction

Mechanical thrombectomy (MT) is the standard of care for acute ischemic stroke due to large vessel occlusion; however, its application to patients with preexisting moderate‐to‐severe disability (modified Rankin Scale [mRS] score 3–5) remains controversial [1, 2, 3, 4]. Several observational studies have explored outcomes in patients with preexisting disability, typically defining success of MT as a return to their baseline mRS score [1, 2, 3, 4]. Yet, this approach assumes that the mRS adequately captures what constitutes a meaningful outcome for a patient who is already living with significant disability.

Older age is tightly linked to preexisting functional dependence in contemporary MT practice. In a multicenter Japanese registry of MT in patients aged ≥ 85 years, 38.6% had substantial pre‐stroke disability (mRS 3–5), underscoring that this population is not uncommon in the “oldest‐old” [5]. This finding emphasizes the need for patient‐centered outcome measures that can detect preserved or regained functional domains related to dignity and participation.

Relying solely on the mRS has important clinical limitations because the scale is heavily weighted toward mobility and often fails to capture subtle but important improvements in quality of life (QOL) [6, 7]. For example, two patients may both be assigned an mRS score of 4: one retains oral intake and meaningful communication with family, whereas the other is largely noncommunicative and depends on enteral nutrition. These critical distinctions, which clinicians often perceive as a worthwhile intervention, are inadequately reflected by the mRS [8, 9]. This measurement gap highlights an unmet need for an outcome scale that aligns more closely with patient‐centered domains such as communication, oral intake, and social engagement—elements that patients and families may prioritize for maintaining dignity, participation, and QOL [10, 11].

ADL‐based scales such as the Barthel Index and Functional Independence Measure (FIM) offer a more detailed view of daily care needs beyond the mRS and have been used as secondary outcomes in MT studies [12, 13, 14]. However, in acute MT practice—particularly for patients with substantial pre‐stroke disability—these instruments may be limited by administration burden and floor effects in low‐functioning ranges. This gap motivated us to develop a brief, bedside‐feasible, patient‐centered measure to capture patient‐centered functional status that the mRS may miss.

The prognostic significance of pre‐stroke disability etiology also remains underinvestigated, as most prior studies have treated pre‐stroke mRS as a single ordinal variable.

Here, we introduce the SMILE‐Stroke score, a clinically derived measure for MT‐treated patients with pre‐stroke mRS scores of 3–5, and evaluate its feasibility, reliability, validity, relationship with conventional outcomes, pre‐stroke SMILE‐Stroke scores, and pre‐stroke disability etiology.

## 2 Methods

### 2.1 Study Design and Population

We conducted a single‐center retrospective cohort study of consecutive adult patients with acute ischemic stroke who underwent MT at Nagoya City University Hospital between January 2015 and July 2025 and had a pre‐stroke mRS score of 3–5. This restriction was chosen because the SMILE‐Stroke score was designed to address an outcome‐assessment gap in patients with preexisting moderate‐to‐severe disability, for whom conventional favorable outcomes such as mRS 0–2 are often unattainable. Pre‐stroke mRS was defined as functional status immediately before the index stroke and was determined from medical records, referral documents, and information from family members or care facilities when available. The study was approved by the Institutional Ethics Committee of Nagoya City University Hospital (Approval Number 60‐25‐0113).

### 2.2 The SMILE‐Stroke Score: A Novel Outcome Measure

To capture patient‐centered outcomes not fully represented by the mRS, we developed a clinically derived 10‐point composite score, the SMILE‐Stroke score. The five domains—Speech/communication, Mobility, Ingestion/oral intake, Lavatory/toileting, and Engagement—were selected from the stroke and serious‐illness literature because they represent functions closely related to dignity, care burden, participation, and QOL [9, 10, 15, 16, 17, 18, 19].

The SMILE‐Stroke score comprises Speech (0–2 points), Mobility (0–2 points), Ingestion (0, 2, or 3 points), Lavatory (0–2 points), and Engagement (0–1 point); detailed scoring criteria are shown in Table 1. The weighting was pragmatically determined by stroke neurologists based on bedside feasibility and clinical interpretability. Ingestion/oral intake was assigned 0, 2, or 3 points to distinguish tube or parenteral nutrition, fully assisted oral intake, and self‐feeding. Mobility and Lavatory were scored dichotomously using clinically practical thresholds: walking several meters and using a toilet, respectively, with or without assistance. Engagement was defined by observable responses to social or environmental stimuli, such as eye contact, tracking, facial expression, or attention to family conversation or television.

**TABLE 1:** The SMILE‐Stroke score components and criteria.

Domain (range)	Score	Criteria
Speech/communication (0–2)	2	Able to communicate functional daily needs using words or sentences.
	1	Able to communicate basic needs (e.g., “yes/no”).
	0	Unable to communicate.
Mobility (0 or 2)	2	Able to walk several meters, with or without assistance or aids.
	0	Unable to walk (includes standing or sitting only).
Ingestion (0, 2, or 3)	3	Able to eat orally without assistance (supervision or partial help with setup is permitted).
	2	Able to eat orally but requires full assistance.
	0	Unable to take nutrition orally (primary nutrition via tube feeding or parenteral nutrition).
Lavatory (0 or 2)	2	Able to use a toilet, with or without assistance.
	0	Dependent on diapers or an indwelling catheter.
Engagement (0–1)	1	Shows interest in surroundings (e.g., follows conversations, watches television, responds to family with facial expressions).
	0	Shows no interest or response to surroundings.
Total score (0–10)		Favorable outcome: ≥ 4 points

*Note:* The score was assessed based on the patient's best functional state within 48 h prior to discharge. Some domains use noncontinuous scoring: Mobility and Lavatory allow only 0 or 2 points, and Ingestion uses 0, 2, or 3 points.

The discharge score was based on the patient's best functional status within 48 h before discharge. Pre‐stroke SMILE‐Stroke scores were retrospectively assigned using the same criteria based on admission history, rehabilitation records, referral documents, and information from family members or care facilities when available.

### 2.3 Outcome Measures and Predictor Variables

The primary outcome was a favorable SMILE‐Stroke outcome, defined as a discharge SMILE‐Stroke score of ≥ 4. This threshold was prespecified a priori because no single domain can contribute 4 points, making the cutoff dependent on preserved or regained function across at least two domains. The score was designed to assess patient‐centered functional status after treatment and was not intended as a pretreatment triage tool.

Secondary outcomes included discharge mRS, 90‐day mRS, return to baseline pre‐stroke mRS, and discharge Barthel Index. The discharge Barthel Index was obtained from routine records when available, or retrospectively calculated from nursing summaries and medical records. We also assessed pre‐stroke SMILE‐Stroke scores to describe baseline patient‐centered functional status and compared discharge SMILE‐Stroke scores with conventional outcome measures.

Discharge disposition was analyzed both as a dichotomous clinical anchor—home/original residence or rehabilitation hospital versus long‐term care hospital or in‐hospital death—and as a four‐category outcome: home/original residence, rehabilitation hospital, long‐term care hospital, and death. Predictor variables included baseline demographics, admission National Institutes of Health Stroke Scale (NIHSS) and Alberta Stroke Program Early CT Score (ASPECTS), treatment‐related variables, and the etiology of pre‐stroke disability, which was categorized by retrospective chart review into cerebrovascular, neurodegenerative, orthopedic, cardiac, malignant, or other causes.

### 2.4 Statistical Analysis

Patients were dichotomized according to the primary outcome: SMILE‐Stroke score ≥ 4 versus < 4. Continuous variables are presented as medians and interquartile ranges (IQR) and were compared using the Mann–Whitney *U* test. Categorical variables are presented as numbers and percentages and were compared using Fisher's exact test. Because of the small sample size and exploratory nature of the study, no multivariable analysis or adjustment for multiple comparisons was performed.

The distribution of total SMILE‐Stroke scores was described across the full 0–10 range. The prespecified cutoff of ≥ 4 was evaluated against discharge disposition as an external clinical anchor, and sensitivity analyses were performed using alternative cutoffs of ≥ 3 and ≥ 5. Receiver operating characteristic analysis was performed using the continuous total SMILE‐Stroke score, and the area under the curve (AUC) was calculated with a bootstrap 95% confidence interval (CI). For the four‐category discharge disposition analysis, SMILE‐Stroke scores were compared using the Kruskal–Wallis test, and ordinal association was assessed using Spearman's rank correlation coefficient.

Spearman's rank correlation coefficients were also calculated for discharge SMILE‐Stroke score versus discharge mRS, 90‐day mRS, and discharge Barthel Index. Pre‐stroke and discharge SMILE‐Stroke scores were compared descriptively using paired plots.

Interrater reliability was assessed by three board‐certified neurologists who independently scored all cases using standardized anchors based on chart, nursing, and therapy notes. Raters were blinded to each other's assessments but not to clinical information, including discharge destination, Barthel Index, mRS, 90‐day outcome, or treatment details. Agreement for the total score was assessed using the intraclass correlation coefficient (ICC), and agreement for the dichotomized outcome was assessed using Fleiss' kappa. For the primary analyses, the final pre‐stroke and discharge SMILE‐Stroke scores were determined by consensus after independent scoring by the three raters; disagreements were resolved through discussion. Interrater reliability was calculated using the initial independent ratings before consensus. Exact binomial 95% CIs were calculated for sensitivity, specificity, predictive values, and accuracy; bootstrap 95% CIs were used for AUC, F1‐score, ICCs, and Fleiss' *κ*.

For one patient with missing 90‐day mRS data, the discharge mRS score was used for imputation. Statistical analyses were performed using EZR and Python version 3.11. A two‐sided *p* < 0.05 was considered statistically significant.

## 3 Results

### 3.1 Patient Population and Outcomes

During the study period, 204 patients underwent MT for acute ischemic stroke at our institution. Of these, 31 patients (15.2%) met the inclusion criteria with a pre‐stroke mRS score of 3–5. The cohort was predominantly composed of patients with pre‐stroke mRS 3 (23/31, 74.2%), followed by mRS 4 (7/31, 22.6%) and mRS 5 (1/31, 3.2%). Pre‐stroke SMILE‐Stroke scores were relatively preserved in many patients (median 10 [IQR, 8–10]), whereas the median discharge SMILE‐Stroke score was 1 [IQR, 0–4.5] (Figures 1A and 2). The median change from pre‐stroke to discharge was −6 [IQR, −10 to −3.5]. Twelve patients (38.7%) achieved the primary favorable outcome (SMILE‐Stroke score ≥ 4), while 19 (61.3%) had a poor outcome.

**FIGURE 1:**
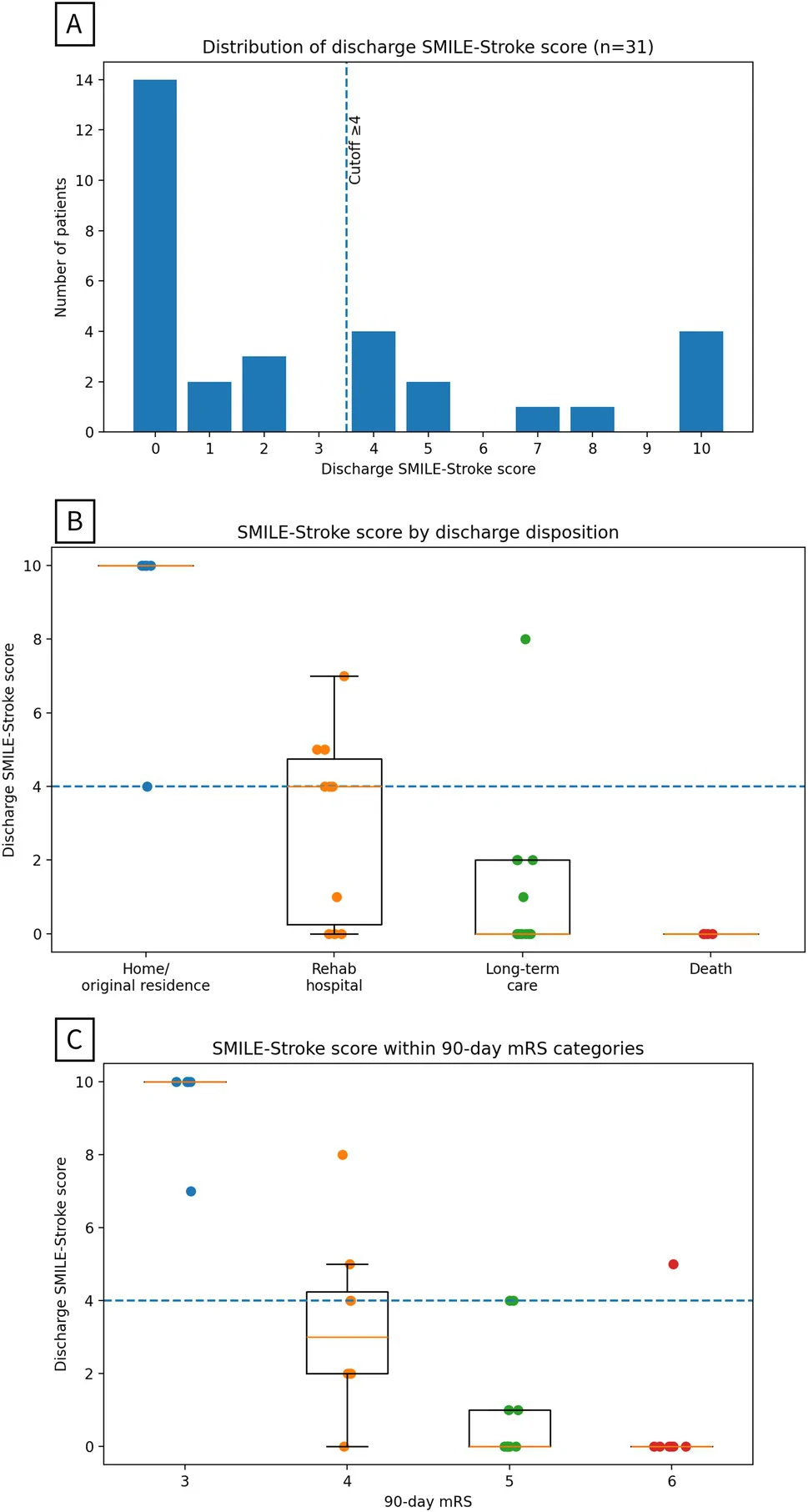
Distribution and clinical anchoring of the SMILE‐Stroke score. (A) Distribution of the total SMILE‐Stroke score at discharge. The vertical dashed line indicates the prespecified favorable‐outcome threshold of ≥ 4 points. (B) SMILE‐Stroke scores according to discharge disposition. Home/original residence and rehabilitation hospital are shown separately to illustrate the graded relationship between the score and real‐world discharge destination. (C) SMILE‐Stroke scores according to 90‐day modified Rankin Scale (mRS) category. The plot illustrates substantial heterogeneity in SMILE‐Stroke scores within conventionally poor mRS categories.

**FIGURE 2:**
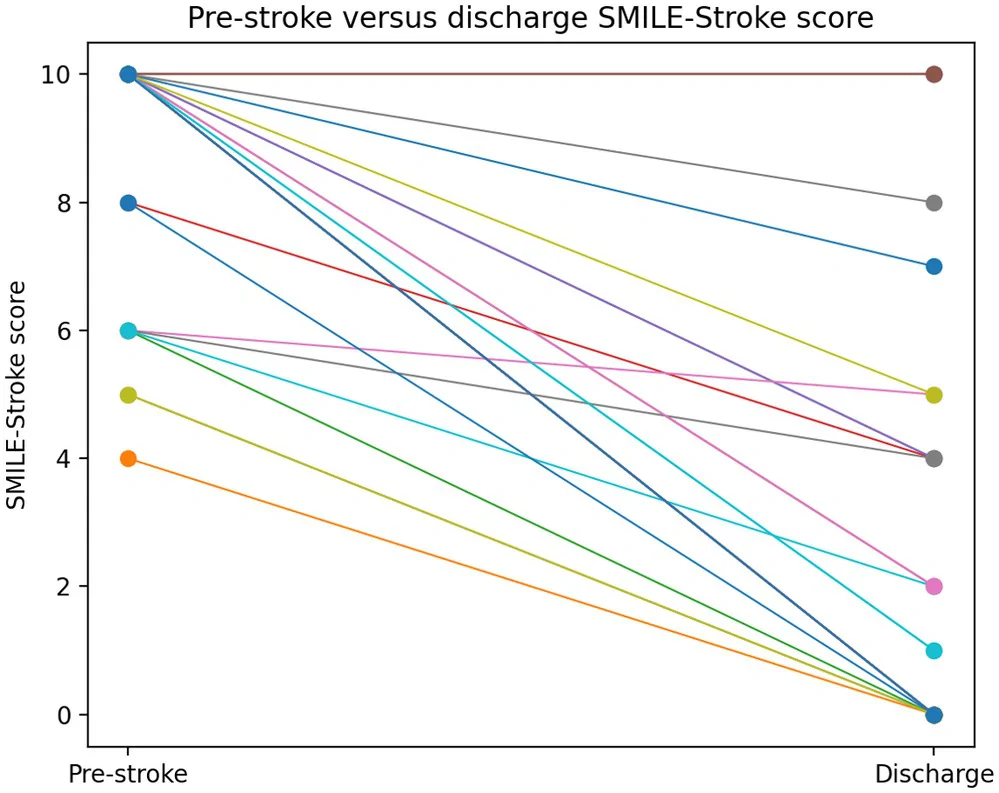
Pre‐stroke and discharge SMILE‐Stroke scores. Paired plot showing pre‐stroke and discharge SMILE‐Stroke scores for each patient. Although most patients had relatively preserved pre‐stroke SMILE‐Stroke scores despite pre‐stroke mRS 3–5, discharge scores varied widely after mechanical thrombectomy, highlighting both patient selection and post‐stroke heterogeneity.

### 3.2 Reliability and Validity of the SMILE‐Stroke Score

For the total score, the ICC indicated almost perfect agreement, with an ICC(2,1) of 0.957 (95% CI, 0.901–0.981) for a single rater and an ICC(2,*k*) of 0.985 (95% CI, 0.964–0.994) for the average of raters. For the dichotomized primary outcome (SMILE‐Stroke score ≥ 4 vs. < 4), Fleiss' kappa (*κ*) was 0.863 (95% CI, 0.691–1.000), which is interpreted as “almost perfect” agreement according to the Landis and Koch criteria [20]. Detailed overall and domain‐level reliability estimates are provided in Tables S1 and S2.

Convergent validity was supported by the strong positive correlation between the total SMILE‐Stroke score and the discharge Barthel Index (Spearman's ρ = 0.863, *p* < 0.001; Figure S1). The SMILE‐Stroke score was also inversely correlated with discharge mRS and 90‐day mRS (Spearman's ρ = −0.774 and −0.739, respectively; Table S3), supporting its association with conventional functional outcomes while allowing additional domain‐level characterization within the same mRS categories.

### 3.3 Comparison of Clinical Characteristics and Outcomes

Baseline characteristics and treatment‐related variables were generally similar between patients with and without a favorable SMILE‐Stroke outcome (Table 2). Patients with a favorable SMILE‐Stroke outcome tended to have higher ASPECTS and shorter door‐to‐puncture time, although these differences were not statistically significant.

**TABLE 2:** Comparison of patient characteristics and outcomes by SMILE‐Stroke score ≥ 4.

	SMILE ≥ 4 (*n* = 12)	SMILE < 4 (*n* = 19)	*p*
Baseline characteristics			
Age (years), median [IQR]	87 [83–88]	87 [84–90]	0.63
Female, *n* (%)	9 (75.0)	13 (68.4)	1
Pre‐stroke mRS, *n* (%)			0.13
3	10 (83.3)	13 (68.4)	
4	1 (8.3)	6 (31.6)	
5	1 (8.3)	0 (0)	
Acute findings			
NIHSS on admission, median [IQR]	20 [17–26]	22 [18–28]	0.68
ASPECTS, median [IQR]	10 [9–10]	8 [8–10]	0.06
Anterior circulation, *n* (%)	12 (100)	18 (94.7)	1
Treatment			
Door‐to‐puncture time (min), median [IQR]	84 [46–116]	115 [82–142]	0.09
tPA administration, *n* (%)	6 (50.0)	10 (52.6)	1
Successful recanalization (mTICI ≥ 2b), *n* (%)	12 (100)	17 (89.5)	0.51
Outcomes			
Discharge destination, *n* (%)			< 0.001*
Home/original residence or rehabilitation hospital	11 (91.7)	4 (21.1)	
Long‐term care or death	1 (8.3)	15 (78.9)	
Barthel Index at discharge, median [IQR]	10 [5–35]	0 [0–0]	< 0.001*
mRS at 90 days, *n* (%)			0.006*
3	5 (41.7)	0 (0)	
4	4 (33.3)	4 (21.1)	
5	2 (16.7)	9 (47.4)	
6	1 (8.3)	6 (31.6)	
Return to baseline mRS, *n* (%)	6 (50.0)	1 (5.3)	0.007*

*Note:* Statistical analysis: continuous variables were compared using the Mann–Whitney *U* test. Categorical variables were compared using Fisher's exact test.

Abbreviations: ASPECTS, Alberta Stroke Program Early CT Score; IQR, interquartile range; mRS, modified Rankin Scale; mTICI, modified Thrombolysis in Cerebral Infarction; NIHSS, National Institutes of Health Stroke Scale; tPA, tissue plasminogen activator.

* *p* < 0.05.

Patients with a SMILE‐Stroke score of ≥ 4 had significantly higher discharge Barthel Index scores (median 10 [IQR, 5–35] vs. 0 [IQR, 0–0], *p* < 0.001), more favorable 90‐day mRS distributions (*p* = 0.006), and a higher rate of returning to baseline pre‐stroke mRS (50.0% vs. 5.3%, *p* = 0.007). They were also more frequently discharged to home/original residence or a rehabilitation hospital than patients with SMILE‐Stroke scores < 4 (91.7% vs. 21.1%, *p* < 0.001).

### 3.4 Clinical Validation of the SMILE‐Stroke Cutoff Using Discharge Disposition

Of the 31 patients, 15 were discharged to home/original residence or transferred to a rehabilitation hospital, whereas 16 were transferred to a long‐term care hospital or died during hospitalization. Total SMILE‐Stroke scores were significantly higher in the home/original residence or rehabilitation hospital group than in the long‐term care hospital or death group (median 4.0 [IQR, 2.5–8.5] vs. 0.0 [IQR, 0–1.25], *p* = 0.001). Using the prespecified cutoff of ≥ 4 points to identify discharge to home/original residence or rehabilitation hospital yielded good discrimination, with an accuracy of 0.84, sensitivity of 0.73, specificity of 0.94, and F1‐score of 0.81. The AUC based on the continuous SMILE‐Stroke score was 0.82 (Figure S2). Sensitivity analyses using alternative cutoffs of ≥ 3 and ≥ 5, including 95% CIs, are shown in Table S4. Because no patient had a SMILE‐Stroke score of 3, the performance of the ≥ 3 and ≥ 4 cutoffs was identical, whereas the ≥ 5 cutoff showed lower sensitivity.

When discharge disposition was separated into four categories, SMILE‐Stroke scores showed a graded association with discharge destination. SMILE‐Stroke scores differed significantly across the four discharge disposition categories (Kruskal–Wallis *p* = 0.0012; Table 3). When discharge disposition was treated as an ordinal variable from home/original residence to death, the SMILE‐Stroke score showed a significant graded association with discharge disposition (Spearman's ρ = −0.699, *p* < 0.001).

**TABLE 3:** SMILE‐Stroke scores according to discharge disposition.

Discharge disposition	*n*	SMILE median [IQR]	SMILE ≥ 4
Home/original residence	5	10 [10–10]	5/5 (100%)
Rehabilitation hospital	10	4 [0.5–4.5]	6/10 (60.0%)
Long‐term care hospital	12	0 [0–2]	1/12 (8.3%)
Death	4	0 [0–0]	0/4 (0%)
Overall	31	1 [0–4.5]	12/31 (38.7%)

*Note:* Data are shown as median [IQR] or number (%). Discharge disposition was categorized into four groups: home/original residence, rehabilitation hospital, long‐term care hospital, and death. SMILE‐Stroke scores differed significantly across discharge disposition categories (Kruskal–Wallis test, *p* = 0.0012). When discharge disposition was treated as an ordinal variable from home/original residence to death, the SMILE‐Stroke score showed a significant graded association with discharge disposition (Spearman's *ρ* = −0.699, *p* < 0.001).

Abbreviation: IQR, interquartile range.

### 3.5 Discordance With Conventional mRS Outcomes

A key finding was the discordance between the SMILE‐Stroke score and conventional mRS outcomes at the individual‐patient level (Figure 1C). Among the 19 patients who would traditionally be classified as having a poor outcome based on a 90‐day mRS score of 4–5, 6 patients (31.6%) achieved a favorable SMILE‐Stroke outcome. Similarly, among the 24 patients who did not return to their pre‐stroke mRS, 6 patients (25.0%) were classified as having a favorable SMILE‐Stroke outcome. These findings suggest that the SMILE‐Stroke score captures clinically relevant heterogeneity within conventionally poor mRS categories rather than simply duplicating mRS‐based outcome classification.

To illustrate this discordance, we describe two patients who both had a 90‐day mRS score of 4. Patient A, who had a pre‐stroke mRS score of 3, regained modified oral intake and simple verbal communication after MT, corresponding to a SMILE‐Stroke score of 8 and a Barthel Index of 10. In contrast, Patient B, who had a pre‐stroke mRS score of 4, remained tube‐fed and diaper‐dependent with only yes/no responses, yielding a SMILE‐Stroke score of 2 and a Barthel Index of 0. These cases are illustrative examples showing how patients assigned to the same mRS category may differ substantially in communication, oral intake, toileting, and engagement; they should not be interpreted as independent evidence of the validity of the SMILE‐Stroke score.

Finally, one patient who died by 90 days (mRS 6) had met the criteria for a favorable SMILE‐Stroke outcome at discharge and was transferred to a rehabilitation hospital. This case highlights that the discharge SMILE‐Stroke score reflects patient‐centered functional status at discharge and may capture short‐term preservation or recovery of meaningful domains, but it does not replace longer‐term outcomes such as 90‐day survival or mRS.

### 3.6 Etiology of Pre‐Stroke Disability and Outcomes

In exploratory analyses, favorable SMILE‐Stroke outcome rates varied numerically according to the etiology of pre‐stroke disability, but no statistically significant association was observed (overall *p* = 0.59; Figure S3). The highest favorable outcome rate was observed in patients with prior cerebrovascular disease (4/7, 57.1%), whereas rates were lower in other etiologic subgroups. Because of the small number of patients in each subgroup, these findings were considered hypothesis‐generating.

## 4 Discussion

We developed SMILE‐Stroke as a brief, patient‐centered outcome measure for MT‐treated patients with pre‐stroke disability. It showed excellent reliability, strong correlation with the Barthel Index, graded association with discharge disposition, and heterogeneity within poor mRS categories. Findings should be interpreted as applying primarily to selected patients with preserved pre‐stroke patient‐centered domains.

### 4.1 Validity and Reliability of the SMILE‐Stroke Score

The SMILE‐Stroke score showed excellent interrater reliability for both the total score and the dichotomized favorable outcome. At the domain level, however, Speech/communication and Engagement showed lower agreement than the other domains (Table S2), consistent with their greater reliance on clinical interpretation. Because domain‐level disagreements were generally limited to adjacent scoring levels and Engagement contributes at most one point, the total score and its dichotomization remained highly reliable. Nonetheless, the operational criteria for these two domains warrant further refinement, and prospective validation with formal rater training is needed.

The strong correlation with the Barthel Index supports convergent validity as a measure of functional status. However, SMILE‐Stroke is not intended to duplicate the Barthel Index. It preserves key ADL‐related domains such as oral intake, mobility, and toileting while adding communication and engagement, which are central to dignity and social participation but are not directly captured by the Barthel Index. Its association with discharge disposition further supports its clinical relevance, although discharge destination should be regarded as an external clinical anchor rather than a definitive validation standard.

### 4.2 Clinical Relevance of the Prespecified Cutoff

We prespecified a SMILE‐Stroke score of ≥ 4 as the favorable‐outcome threshold based on the structure and clinical intent of the scale. Because no single domain can contribute 4 points by itself, this threshold requires preserved or regained function across at least two domains. Thus, the cutoff was intended to represent a clinically meaningful level of patient‐centered functional status rather than a statistically optimized threshold.

The external anchoring analyses supported the clinical interpretability of this cutoff: a score of ≥ 4 was associated with discharge to home/original residence or rehabilitation hospital, and SMILE‐Stroke scores showed a graded relationship across discharge disposition categories. Sensitivity analyses showed that the ≥ 5 cutoff reduced sensitivity, whereas the ≥ 3 and ≥ 4 cutoffs performed identically because no patient had a score of 3. Therefore, the present data support ≥ 4 as a clinically prespecified and empirically reasonable threshold, but do not establish it as an optimal cutoff.

### 4.3 Discordance With mRS: Capturing Patient‐Centered Heterogeneity

The SMILE‐Stroke score revealed clinically relevant heterogeneity within conventional mRS categories. In our cohort, 31.6% of patients with a 90‐day mRS score of 4–5 and 25.0% of those who did not return to baseline mRS achieved a favorable SMILE‐Stroke outcome. These findings do not imply that the mRS is inappropriate or that SMILE‐Stroke should replace it. Rather, they suggest that the mRS may compress meaningful differences in communication, oral intake, toileting, and engagement into the same category of severe disability [6, 7, 8].

This distinction is particularly important in patients with pre‐stroke disability, for whom full independence is often unrealistic. Preservation or recovery of specific domains—such as communicating with family, eating orally, using a toilet with assistance, or showing social responsiveness—may influence dignity, care goals, and family‐perceived treatment benefit. The illustrative cases, in which two patients with the same 90‐day mRS score of 4 had SMILE‐Stroke scores of 8 and 2, demonstrate the type of domain‐level heterogeneity that motivated the score, but should not be interpreted as independent evidence of validity. Similarly, the patient who achieved a favorable discharge SMILE‐Stroke score but died by 90 days highlights that the score reflects discharge‐time patient‐centered functional status and does not replace survival or 90‐day mRS.

### 4.4 Comparison With Existing Functional Scales

Conventional functional scales remain essential but have limitations in patients with pre‐stroke disability. The mRS is simple and widely used, but as a global disability scale it may compress differences in communication, oral intake, toileting, and engagement into the same severe‐disability category [6, 7, 9]. The Barthel Index captures basic ADL but does not directly assess communication or engagement [13, 14, 21, 22], whereas the FIM and patient‐reported measures are more comprehensive but less consistently available in retrospective acute MT cohorts [7, 9, 12, 23]. Accordingly, SMILE‐Stroke is positioned as a brief adjunct that preserves key ADL‐related domains while adding communication and engagement.

### 4.5 Pre‐Stroke Disability Etiology as an Exploratory Factor

Favorable outcomes were numerically more frequent in patients with prior cerebrovascular disease, possibly reflecting preserved pre‐stroke domains. However, subgroup sizes were small and differences were nonsignificant; these findings are hypothesis‐generating.

### 4.6 Limitations

This study has several limitations. First, it was a retrospective, single‐center study with a small sample size, limiting statistical power and generalizability. Patients with pre‐stroke mRS 4–5 were underrepresented, and many patients had preserved pre‐stroke SMILE‐Stroke domains despite pre‐stroke mRS scores of 3–5. Thus, the findings apply primarily to selected MT‐treated patients in whom patient‐centered domains were preserved before stroke. Such selection may reflect real‐world MT decision‐making that incorporates communication, oral intake, toileting, and engagement, explicitly or implicitly, beyond the mRS alone; however, because untreated patients were not included, this interpretation remains speculative. The SMILE‐Stroke score was not evaluated in patients with pre‐stroke mRS scores of 0–2, for whom conventional favorable outcomes such as mRS 0–2 remain appropriate primary endpoints.

Second, the score was developed and evaluated in the same cohort. Although the cutoff of ≥ 4 was prespecified and supported by discharge disposition analyses, it has not been externally validated, and the ≥ 3 and ≥ 4 cutoffs performed identically because no patient had a score of 3. Third, scoring was based on retrospective chart review. Raters were blinded to each other's assessments but not to clinical information, which may have introduced information bias, and pre‐stroke SMILE‐Stroke scores may have been misclassified. Finally, discharge disposition can be influenced by healthcare‐system and social factors, and the discharge SMILE‐Stroke score does not replace longer‐term outcomes such as survival, 90‐day mRS, or QOL. Because this study included only MT‐treated patients and had no untreated control group, the findings should not be interpreted as evidence that MT improves SMILE‐Stroke outcomes. Prospective multicenter studies with blinded assessment, patient and family input, and external validation are needed.

## 5 Conclusion

The SMILE‐Stroke score is a brief, patient‐centered outcome measure for MT‐treated patients with pre‐stroke disability. In this single‐center retrospective cohort, it showed excellent interrater reliability, strong correlation with the Barthel Index, and meaningful associations with discharge disposition and mRS outcomes. The score captured heterogeneity within poor mRS categories by identifying preserved or regained domains such as communication, oral intake, toileting, mobility, and social engagement. SMILE‐Stroke may therefore serve as a pragmatic adjunct to the mRS in selected patients with preexisting disability. Prospective multicenter validation with blinded assessment and patient or family input is needed.

## Author Contributions

H. Inoue analyzed the data and drafted the manuscript. Y. Nakamura and M. Oomura contributed to study design and critically revised the manuscript. Y. Uchida contributed to statistical analysis, interpretation of data, and critical revision of the manuscript for important intellectual content. Y. Nishikawa, S. Kusui, A. Taishaku, Y. Nozaki, T. Yamanaka, T. Shibata, Y. Inoue, Y. Taniguchi, Y. Madokoro, T. Sato, T. Fujioka, M. Mizuno, and S. Kawashima contributed to data acquisition/interpretation and reviewed the manuscript. M. Mase and N. Matsukawa supervised the study and approved the final manuscript.

## Funding

The authors have nothing to report.

## Ethics Statement

This study was conducted following the approval of the Institutional Ethics Committee of Nagoya City University Hospital (Approval Number 60‐25‐0113).

## Consent

Individual informed consent was waived; an opt‐out process was provided on the institutional website.

## Conflicts of Interest

The authors declare no conflicts of interest.

## Supporting information


**Figure S1:** Scatter plot showing the relationship between the discharge SMILE‐Stroke score and the Barthel Index at discharge. Larger markers indicate overlapping observations. The horizontal line denotes the prespecified favorable‐outcome threshold (SMILE‐Stroke score ≥ 4). Spearman's *ρ* was 0.863 (*p* < 0.001).
**Figure S2:** Receiver operating characteristic curve for the continuous discharge SMILE‐Stroke score. The curve discriminates favorable discharge disposition (home/original residence or rehabilitation hospital) from long‐term care hospital or death. The area under the curve was 0.821 (bootstrap 95% confidence interval [CI], 0.66–0.95).
**Figure S3:** Achievement rate of a favorable outcome (SMILE‐Stroke score ≥ 4) according to the principal etiology of pre‐stroke disability. These subgroup analyses were exploratory, and the results should be interpreted cautiously because of the small number of patients in each etiologic category. No statistically significant association was observed across categories (overall *p* = 0.59).
**Table S1:** Overall interrater reliability of the SMILE‐Stroke score.
**Table S2:** Interrater reliability of the SMILE‐Stroke score by domain.
**Table S3:** Associations between the discharge SMILE‐Stroke score and conventional functional outcomes.
**Table S4:** Sensitivity analysis of alternative SMILE‐Stroke score cutoffs for favorable discharge disposition.

## Data Availability

The data that support the findings of this study are available on request from the corresponding author. The data are not publicly available due to privacy or ethical restrictions.
