# Supplementary material for: Beyond the Rankin Scale: A Patient‐Centered Score for Meaningful Recovery After Mechanical Thrombectomy in Older Adults With Pre‐Stroke Disability
Source: Geriatr Gerontol Int. 2026 Oct 16;26(10):e70874. doi: 10.1111/ggi.70874 (PMC13675113; doi:10.1111/ggi.70874)
Supplement: Supplementary file 1 [file GGI-26-0-s001.docx]

**Supplementary Material**

**Supplementary Figure S1. Scatter plot showing the relationship between the discharge SMILE-Stroke score and the Barthel Index at discharge.**

Larger markers indicate overlapping observations. The horizontal line denotes the prespecified favorable-outcome threshold (SMILE-Stroke score ≥4). Spearman’s ρ was 0.863 (P < 0.001).

**Supplementary Figure S2. Receiver operating characteristic curve for the continuous discharge SMILE-Stroke score.**

The curve discriminates favorable discharge disposition (home/original residence or rehabilitation hospital) from long-term care hospital or death. The area under the curve was 0.821 (bootstrap 95% confidence interval [CI], 0.66–0.95).

**Supplementary Figure S3. Achievement rate of a favorable outcome (SMILE-Stroke score ≥4) according to the principal etiology of pre-stroke disability.**

These subgroup analyses were exploratory, and the results should be interpreted cautiously because of the small number of patients in each etiologic category. No statistically significant association was observed across categories (overall P = 0.59).

**Supplementary Table S1. Overall interrater reliability of the SMILE-Stroke score**

| **Measure** | **Estimate (95% CI)** | **Details** |
| --- | --- | --- |
| Total SMILE-Stroke score | ICC(2,1) = 0.957 (0.901–0.981) | Single-rater absolute agreement |
| Total SMILE-Stroke score | ICC(2,k) = 0.985 (0.964–0.994) | Average of three raters |
| Favorable outcome classification | Fleiss’ κ = 0.863 (0.691–1.000) | SMILE-Stroke score ≥4 vs. <4 |
| Favorable outcome classification | 28/31 (90.3%) | Complete agreement among all three raters |

ICC(2,1), two-way random-effects, single-measure, absolute-agreement intraclass correlation coefficient; ICC(2,k), average-measure intraclass correlation coefficient. Confidence intervals were estimated using bootstrap resampling. CI, confidence interval.

**Supplementary Table S2. Interrater reliability of the SMILE-Stroke score by domain**

| **Domain** | **ICC(2,1) (95% CI)** | **Fleiss’ κ (95% CI)** | **Exact agreement** |
| --- | --- | --- | --- |
| Speech | 0.813 (0.687–0.896) | 0.585 (0.400–0.752) | 19/31 (61.3%) |
| Mobility | 0.831 (0.611–1.000) | 0.827 (0.603–1.000) | 28/31 (90.3%) |
| Ingestion | 0.949 (0.864–0.993) | 0.828 (0.668–0.958) | 27/31 (87.1%) |
| Lavatory | 0.860 (0.562–1.000) | 0.856 (0.552–1.000) | 29/31 (93.5%) |
| Engagement | 0.493 (0.248–0.706) | 0.484 (0.225–0.698) | 19/31 (61.3%) |

Three board-certified neurologists independently scored all 31 patients. Domain-specific analyses were exploratory. Confidence intervals were estimated using bootstrap resampling. ICC(2,1), two-way random-effects, single-measure, absolute-agreement intraclass correlation coefficient; CI, confidence interval.

**Supplementary Table S3. Associations between the discharge SMILE-Stroke score and conventional functional outcomes**

| **Comparison** | **Spearman’s ρ** | **P value** |
| --- | --- | --- |
| Discharge mRS vs. discharge SMILE-Stroke score | −0.774 | <0.001 |
| 90-day mRS vs. discharge SMILE-Stroke score | −0.739 | <0.001 |
| Discharge BI vs. discharge SMILE-Stroke score | 0.863 | <0.001 |

Correlations were calculated using Spearman’s rank correlation coefficient. mRS, modified Rankin Scale; BI, Barthel Index.

**Supplementary Table S4. Sensitivity analysis of alternative SMILE-Stroke score cutoffs for favorable discharge disposition**

| **Cutoff** | **Sensitivity, % (95% CI)** | **Specificity, % (95% CI)** | **PPV, % (95% CI)** | **NPV, % (95% CI)** | **Accuracy, % (95% CI)** | **F1 score (95% CI)** | **Youden index, %** |
| --- | --- | --- | --- | --- | --- | --- | --- |
| ≥3 | 73.3 (44.9–92.2) | 93.8 (69.8–99.8) | 91.7 (61.5–99.8) | 78.9 (54.4–93.9) | 83.9 (66.3–94.5) | 0.815 (0.61–0.96) | 67.1 |
| ≥4 | 73.3 (44.9–92.2) | 93.8 (69.8–99.8) | 91.7 (61.5–99.8) | 78.9 (54.4–93.9) | 83.9 (66.3–94.5) | 0.815 (0.61–0.96) | 67.1 |
| ≥5 | 46.7 (21.3–73.4) | 93.8 (69.8–99.8) | 87.5 (47.3–99.7) | 65.2 (42.7–83.6) | 71.0 (52.0–85.8) | 0.609 (0.32–0.81) | 40.4 |

Favorable discharge disposition was defined as discharge to home/original residence or transfer to a rehabilitation hospital; unfavorable disposition was defined as transfer to a long-term care hospital or death. Exact binomial 95% CIs were calculated for sensitivity, specificity, PPV, NPV, and accuracy. The 95% CI for the F1 score was estimated using bootstrap resampling. The area under the receiver operating characteristic curve, calculated using the continuous total SMILE-Stroke score, was 0.821 (bootstrap 95% CI, 0.66–0.95). The ≥3 and ≥4 cutoffs yielded identical results because no patient had a discharge SMILE-Stroke score of 3. CI, confidence interval; PPV, positive predictive value; NPV, negative predictive value.
